# Persistent Paralysis of the Leg

**Published:** 1914-05

**Authors:** 


PERSISTENT PARALYSIS OF THE LEG. Ingenious treatment by a peasant, Chambon, *Jour de Med. de Bordeaux,* November 30, 1913. The patient, after being incapacitated for six months after sciatica, the leg being atrophied and the paralysis interfering with the elevation of the foot in walking, tied one end of a cord to his coat button and looped the other end about his instep. By raising the foot by pulling on the cord, so as to raise the foot at each step, and using a cane in the opposite hand, he managed to walk fairly well.

